# Discordance among skeletal maturity indicators: sources, interpretation, and clinical implications

**DOI:** 10.3389/fped.2026.1970006

**Published:** 2026-09-23

**Authors:** Yongfu Zhou, Tianrui Shi, Lixin Tang, Yikai Li, Siyuan Xie

**Affiliations:** 1 Nanfang Hospital, Southern Medical University, Guangzhou, Guangdong, China; 2 Linhe Street Community Health Service Center, Guangzhou, Guangdong, China; 3 School of Traditional Chinese Medicine, Southern Medical University, Guangzhou, Guangdong, China; 4 The Third Affiliated Hospital, Southern Medical University, Guangzhou, Guangdong, China; 5 Department of Sports Medicine, The First Affiliated Hospital, Guangdong Provincial Key Laboratory of Speed Capability, Guangzhou Key Laboratory of Precision Orthopedics and Regenerative Medicine, School of Medicine, Jinan University, Guangzhou, China

**Keywords:** adolescent idiopathic scoliosis, bone age, growth plate, regional developmental differences, remaining growth, skeletal maturity

## Abstract

Bone age and regional skeletal maturity staging are widely used to identify growth stage, estimate remaining growth, and support orthopedic treatment decisions in children and adolescents. However, skeletal maturity indicators differ in the regions examined, developmental features observed, scoring rules applied, and reference standards used. Consequently, the same child may receive different age estimates or maturity classifications when a different bone group, anatomical region, imaging modality, or assessment method is selected. This narrative review synthesizes evidence from within-image bone-group comparisons, contemporaneous cross-regional assessments, disease and local-intervention studies, mechanistic research, and validation against clinical outcomes. We organize the evidence according to the information represented by each measure and the outcome against which it has been evaluated. Discordance may arise from the imaging and assessment process itself, including modality, scoring method, method version, and population-specific reference standards. It may also reflect regional differences in growth plate responses to systemic signals, prior growth history, and local mechanical conditions. Animal, cellular, and tissue studies support the biological plausibility of site-specific responses, but they do not quantify the magnitude or sequence of regional differences in children. The clinical value of a skeletal maturity indicator must therefore be established separately for the outcome it is intended to inform, such as peak height velocity, adult height, actual growth of a target bone segment, scoliosis progression, or treatment outcome. High correlation between indicators or a small mean difference at the group level does not ensure close agreement within an individual child. Future research should prioritize contemporaneous paired assessments, longitudinal multiregional cohorts, reporting of individual-level differences and stage reclassification, and multicenter external validation. These approaches can help clarify how discordance should be interpreted and whether its magnitude is sufficient to alter a specific clinical judgment.

## 1 Introduction

Bone age and regional skeletal maturity indicators are commonly used to identify growth stage, estimate remaining growth, predict adult height, and support orthopedic treatment decisions in children and adolescents (1, 2). Established methods are valuable in these settings, but they do not observe the same anatomy or derive their outputs in the same way. Some compare the overall hand-wrist appearance with an atlas, some assign scores to individual bones, and others stage ossification, morphologic change, or fusion at the elbow, spine, pelvis, or knee. Although all of these approaches describe skeletal development, they do not necessarily capture the same developmental information or answer the same clinical question.

Discordant results have been documented in two broad settings. Different bone groups or skeletal regions in the same child may yield different assessments, and the result from a single image may change when another method or version is applied (3–5). Such differences may originate from the region observed, imaging modality, scoring procedure, or reference standard, but may also include genuine regional variation in development. Their source must be considered before deciding whether they alter growth prediction, risk classification, or treatment timing.

This review first defines the information represented by commonly used indicators and then synthesizes direct evidence of discordance and its methodological and biological sources. The evidence is subsequently evaluated in relation to peak height velocity (PHV), adult height, remaining growth of a target bone segment, and orthopedic applications, with adolescent idiopathic scoliosis (AIS) serving as a clinically informative example.

## 2 Literature search, evidence selection, and appraisal

### 2.1 Literature search and evidence selection

PubMed, Scopus, and the Web of Science Core Collection were searched from database inception to 27 July 2026. Six prespecified search modules covered: (1) direct comparisons between skeletal regions or maturity indicators; (2) differences between carpal and radius–ulna–short-bone maturation; (3) regional developmental timing and growth plate mechanisms; (4) validation against growth-related or clinical outcomes; (5) disease-, environment-, or intervention-related regional differences; and (6) regional, multiregional, or opportunistic imaging methods. No database filters were applied for publication year, language, study design, population, or publication type. Grey literature was not systematically searched. The complete database-specific strategies, search fields, dates, and module-level yields are provided in Supplementary Table S1.

Eligible sources included human studies that compared two or more skeletal regions, bone groups, maturity indicators, scoring systems, or method versions; characterized regional ossification, physeal closure, or maturation timing; or evaluated a maturity indicator against peak height velocity, final height, remaining global or segmental growth, scoliosis behavior, or treatment outcomes. Studies of healthy children and adolescents and populations with growth-related conditions were eligible. Experimental animal, laboratory, and human-tissue studies were considered when they provided site-specific mechanistic evidence relevant to growth plate maturation. Authoritative methodological publications, reviews, and clinical guidance documents were used to describe established assessment systems and provide clinical context. Sources were not retained when they addressed only the reliability or chronological-age accuracy of a single method without regional or outcome-linked relevance, focused exclusively on forensic age estimation, or provided insufficient or substantially duplicative evidence. The detailed eligibility criteria are provided in Supplementary Table S2.

The 18 database-by-module searches yielded 8,752 records before deduplication across databases and search modules. After 4,271 duplicate records were removed, 4,481 unique records underwent manual title-and-abstract screening against the predefined eligibility criteria, and 110 were retained for full-text assessment. Two reviewers independently screened all 4,481 unique records by title and abstract, and the same two reviewers independently assessed all 110 full-text candidates. Disagreements at either stage were resolved through discussion with a third reviewer. Of the 110 database-derived full-text candidates, 62 were retained after full-text eligibility and relevance assessment, whereas 48 were excluded. Targeted supplementary searches, source verification, and backward and forward citation tracking were subsequently conducted to verify coverage of foundational methodological studies, relevant guidance documents, key clinical cohorts, and non-radiographic predictors. Sources identified through these procedures were evaluated using the same eligibility criteria. These supplementary procedures contributed 12 additional eligible sources, resulting in a total of 74 sources retained for the final narrative synthesis. The 12 supplementary sources and their routes of identification are listed in Supplementary Table S3. The complete numerical flow and principal reasons for exclusion are shown in Supplementary Figure S1.

### 2.2 Evidence appraisal and synthesis

Given the methodological heterogeneity of the eligible literature and the narrative objective of this review, no formal design-specific risk-of-bias instruments or aggregate quality scores were applied. Instead, studies were interpreted according to their design, temporal framework, within-person or between-group comparability, ability to distinguish anatomical region from the assessment pathway, relevance of the reference outcome, major sources of confounding, and applicability to the question being addressed. These considerations were used to define the appropriate scope of interpretation rather than as formal quality ratings or exclusion thresholds. Representative design features, applicability, and principal limitations are summarized in Tables 1, 2.

**Table 1:** Evidence category, design features, and interpretive scope of representative comparisons across skeletal regions or assessment methods.

Study	Evidence category	Comparison	Main finding	Design features and interpretive scope
Carpenter and Lester, 1993 (3)	I	Carpal, distal radius/ulna, and metacarpal/phalangeal regions on the same hand-wrist image	Carpal bone age was 4–25 months younger than the whole-hand assessment on 77 of 100 images	Within each comparison, child-level developmental state, examination time, image, imaging modality, and the GP atlas framework were held constant. However, different bone groups were matched to region-specific atlas features; therefore, the findings demonstrate within-hand discordance but do not isolate regional biology from assessment and reference effects.
Zhang et al., 2008 (20)	I	TW3-C RUS versus carpal bone age	Mean group difference was near zero; SD of paired differences was about one year in some male age groups	The same hand-wrist image and TW3-C framework were used, reducing variation attributable to examination timing and imaging modality. However, the RUS and carpal assessments used bone-group-specific scoring and age-conversion standards, so biological and method-related contributions remain partly coupled.
Xi and Roche, 1990 (4)	II	Hand-wrist versus knee bone age	Mean absolute difference 0.34–0.87 years; maximum individual difference 1.45–2.99 years	Hand-wrist and knee assessments were paired within the same children at scheduled examinations, reducing between-child and some temporal variation. However, anatomical region, selected maturity features, scoring procedures, and regional reference models all differed; therefore, the study demonstrates cross-regional assessment discordance but cannot isolate a purely regional biological effect.
Aicardi et al., 2000 (26)	II	Multiple hand-wrist and knee methods	Direction of difference reversed between children with delayed and advanced maturation	Within-child comparisons reduced between-child developmental variation, but both anatomical region and assessment method changed in a referred cross-sectional population. The study therefore describes cross-regional discordance but cannot separate regional from methodological effects.
Jang et al., 2022 (28)	II	GP hand-wrist versus Sauvegrain elbow assessment	Mean absolute deviations from chronological age in boys were 1.26 and 0.61 years	The two assessments were obtained in the same children within two months, reducing temporal variation. Because anatomical region, image, and scoring method differed, the observed discrepancy cannot be attributed specifically to regional development; moreover, chronological age was not a longitudinal growth outcome.
Cheung and Cheung, 2021 (60)	II	Risser sign, Sanders staging, and DRU classification	Risser 0 + encompassed Sanders 2–7; systems differed in information on growth velocity and plateau	The maturity systems were evaluated longitudinally in the same AIS cohort, allowing comparison with observed growth behavior. However, the systems assessed different anatomical regions and used different staging rules. The study supports clinically relevant staging discordance rather than isolation of regional biology, and the broad developmental interval represented by Risser 0 + limits one-to-one correspondence with Sanders and DRU stages.
Sun et al., 2025 (27)	II	Knee MRI versus hand-wrist bone age	Knee MRI appeared more mature in 14.1% of adolescents with slowing growth velocity	Participants were evaluated within the same clinical cohort, but anatomical region, imaging modality, atlas or model, and the overall assessment pathway differed. The study demonstrates clinically relevant discordance between two assessment pathways but cannot determine how much is attributable to regional development; the reported endocrine association was also cross-sectional.
Vignolo et al., 1990 (33)	II	Knee RWT versus hand-wrist GP/TW	Indicators from different regions were highly correlated	Several skeletal assessments were obtained in the same participants, reducing between-person variation and allowing comparison across methods. However, anatomical region and regional reference systems remained coupled; the reported correlations describe shared developmental trends but do not quantify the magnitude of within-child disagreement.
Ouyang et al., 2026 (34)	II	Spheno-occipital synchondrosis, hand-wrist, and CVM	Strong associations; kappa about 0.78 for selected classifications	Assessment of the same participants reduced between-person variation. However, anatomical region, imaging acquisition, and classification system differed, and no longitudinal growth outcome was evaluated; therefore, the reported agreement does not isolate regional developmental effects.
Shaw and Herring, 2023 (29)	III	Hand versus hip in LCP disease	Hand age lagged chronological age by 1.4 years; mean hand-hip difference about 1.7 years	Within-child comparison reduced systemic between-person variation. However, anatomical region, assessment method, disease-related hip involvement, and loading remained coupled; therefore, the findings are most informative for disease-context discordance rather than normal regional maturation.
Lee et al., 2017 (30)	III	Bilateral hand assessment in unilateral spastic cerebral palsy	Affected-side bone age and limb lengths were lower; MACS level correlated separately with bone-age and segmental length differences.	The same skeletal region, imaging modality, and GP method were compared bilaterally within each child, holding the systemic developmental background largely constant. This design supports a local contribution to side-to-side differences, although the specific effects of innervation, loading, perfusion, limb use, and disease severity cannot be separated.

Categories I–IV are organizational categories based on study design and are not intended as a hierarchy of evidence quality. Category I comprises same-image comparisons between bone groups; Category II comprises cross-regional comparisons that also change the assessment method or imaging modality; Category III comprises within-person disease or local-intervention comparisons, including bilateral affected-versus-unaffected designs; and Category IV comprises mechanistic animal, laboratory, or human-tissue evidence. The final column identifies factors held constant by each design, factors that remained coupled, and the extent to which the study can distinguish regional developmental effects from differences in the assessment pathway. Category IV evidence is discussed in Section 5.2 and is not included in this table because the table summarizes direct human comparisons. AIS, adolescent idiopathic scoliosis; CVM, cervical vertebral maturation; DRU, distal radius and ulna; GP, Greulich-Pyle; LCP, Legg-Calve-Perthes; RUS, radius, ulna, and short bones; RWT, Roche-Wainer-Thissen; TW, tanner-whitehouse.

**Table 2:** Representative outcome-linked clinical evidence for skeletal maturity indicators.

Clinical question	Representative evidence and reference outcome	Main finding	Applicability and principal limitation
PHV and growth stage	125 premenarchal girls with AIS, 645 serial hand radiographs; longitudinal TOCI validation (62)	PHV occurred at TOCI 5 in 70.1% and at Sanders 3 in 51.4%	Single-center female AIS cohort; concentration within a stage does not establish superiority for every task
AIS staging mismatch and curve progression	Systematic review/meta-analysis and longitudinal AIS cohort (59, 60)	Risser-Sanders discrepancy rate 16.1%; Risser 0 + encompassed Sanders 2–7	Meta-analysis dichotomized stages; specific treatment outcomes require separate validation
AIS brace discontinuation: scoping review	Scoping review of 43 studies (63)	In seven studies, 236/700 patients (34%) progressed >5 degrees over 2–4 years	Not a pooled rate across all 43 studies; brace cessation and weaning protocols were heterogeneous
AIS brace discontinuation: prospective cohort	Prospective cohort of 144 patients (64)	29% progressed >5 degrees after conventional cessation criteria; later Sanders stages/DRU grades were associated with lower risk	Single-center protocol; stages should not be generalized as universal thresholds
VBT	34 patients followed to maturity (65)	Appendicular indicators informed leg-length growth; axial/TOCI indicators differed in information on curve modulation	Small, selected sample; risks of overfitting and limited transportability
Adult-height prediction	Combined hand-wrist and knee model in 173 postmenarchal girls (57)	Fitted-model *R*^2^ = 0.733; cross-validated mean bias near zero; 95% limits of agreement approximately ± 4 cm	Single-center internal validation; external validation and calibration required
Remaining growth around the knee	52 children with limb-length discrepancy (2)	Automated GP produced smaller femoral/tibial prediction errors than chronological age or Sauvegrain staging	Small sample; does not establish GP as optimal for every local task
Local growth risk in ACL care	79 skeletally immature children with ACL injury (66)	Knee MRI bone age differed from chronological age by >1 year in 25.3%	No follow-up to final growth; remaining growth was estimated from age models
Hemiepiphysiodesis	29 patients followed to maturity (67)	Proximal tibial hemiepiphysiodesis after distal phalangeal fusion still resulted in undercorrection	Specific procedure and genu varum population; interpretation requires observed postoperative correction
Limb lengthening	Achondroplasia lengthening cohort (31)	Actual lower-limb growth was 4.9% lower after tibial lengthening and 8.5% lower after sequential tibial and femoral lengthening than in non-lengthened controls; premature closure occurred predominantly at the proximal tibia and distal femur.	Complex intervention; cannot isolate one mechanism or extrapolate to healthy children
Height velocity in idiopathic scoliosis	Prospective multicenter cohort of 254 patients with serial anthropometry, radiographic maturity assessments, and CXM; height velocity as the reference outcome (73)	CXM explained 50% of the variance in height velocity; CXM combined with the Risser sign explained 55%	Multicenter idiopathic scoliosis cohort with a mean follow-up of 12 months; external validation and evidence of incremental clinical decision value are still needed

ACL, anterior cruciate ligament; AIS, adolescent idiopathic scoliosis; DRU, distal radius and ulna; PHV, peak height velocity; TOCI, thumb ossification composite index; VBT, vertebral body tethering.

## 3 What skeletal maturity indicators represent

### 3.1 Skeletal maturation, assessment outputs, and growth outcomes

Skeletal maturation, bone age, maturity stage, and growth outcome are distinct constructs. Skeletal maturation is the biological process through which endochondral ossification progresses, growth plate function declines, and epiphyseal fusion occurs. Bone age is an age estimate derived from an image of a specified region by applying defined assessment rules and a reference standard. A maturity stage is an ordered classification of current developmental status based on local ossification, morphologic change, or fusion (1, 6). By contrast, PHV, adult height, and the actual growth of a target bone segment are longitudinal outcomes that require serial measurement or follow-up to growth completion. Bone age and maturity stage primarily describe the developmental status visible on an image; their relationships with future growth outcomes require separate longitudinal validation in the relevant population. Reproducible classification of an imaging feature is not equivalent to accurate prediction of an individual child's future growth.

### 3.2 Bone age methods expressed in years

One group of methods converts radiographic findings into a bone age expressed in years. The Greulich-Pyle (GP) atlas compares the overall hand-wrist appearance with reference plates (7). The Tanner-Whitehouse (TW) approach assigns maturity stages to individual bones and converts the resulting scores into skeletal maturity scores or bone age estimates. The TW2 and TW3 editions use different reference standards and score-to-age conversions (8, 9). The FELS method combines categorical morphologic features and measurements in a statistical estimate and can report both bone age and its standard error (1). Although all three methods produce an age-based output, they assess different bones and apply different rules and reference data. In the same group of children, differences between TW2 and TW3 estimates for the radius, ulna, and short bones (RUS) and for the carpal bones varied by age and sex (5). Thus, a shared unit does not imply shared developmental information. Comparisons between methods should therefore use paired within-child analyses and examine whether the differences vary across developmental stages.

### 3.3 Regional maturity staging

Other methods classify maturity without assigning a continuous bone age. Common features include epiphyseal capping, apophyseal ossification and fusion, cervical vertebral morphology, and growth plate bridging on magnetic resonance imaging (MRI). Representative regional systems include the Risser sign, based on iliac apophyseal ossification and fusion (10), and Sanders staging, based on epiphyseal capping and fusion in selected hand bones (11). The distal radius and ulna (DRU) classification evaluates distal radial and ulnar epiphyseal maturation (12), whereas cervical vertebral maturation (CVM) methods principally assess the morphology of C2-C4 (13, 14). Stages generally follow an ordered sequence, but adjacent stages do not necessarily occupy equal time intervals. The Sauvegrain elbow method has good interobserver reliability, although the original system may omit intermediate morphologies (15). Both Sauvegrain staging and GP hand-wrist assessment can be reproducible while their paired estimates differ by more than one year (16). Regional stages should therefore be interpreted first as descriptions of the current status of the region examined. When several stages are collapsed into ‘mature’ and ‘immature’ categories, agreement also depends on the chosen threshold and the stage distribution of the sample. Relationships with PHV or growth remaining in another skeletal segment still require longitudinal validation.

### 3.4 Growth-stage assessment and prediction of remaining growth

Locating a child before, near, or after PHV is related to, but different from, predicting how much growth remains. In historical longitudinal data, RUS bone age was more closely aligned with the timing of PHV than carpal bone age (17). For prediction of growth around the knee, however, an automated GP assessment produced smaller femoral and tibial prediction errors than chronological age or Sauvegrain staging (2). Local proximal tibial morphology also showed predictive value, whereas more complex models for timing epiphysiodesis did not necessarily outperform simpler alternatives (18, 19). Evaluation of an indicator must therefore begin with the intended outcome: identifying the current growth stage and predicting the amount of remaining growth require different longitudinal reference outcomes. Table 3 summarizes the observations, outputs, and appropriate validation targets of major indicator types.

**Table 3:** Observations, outputs, and validation targets of skeletal maturity indicators.

Indicator type	Observation and representative methods	Output	Question addressed and appropriate validation
A. Skeletal maturity assessment			
Overall atlas matching	Overall hand-wrist appearance compared with reference plates, e.g., GP (7)	Bone age in years	Describes overall hand-wrist development; prediction requires validation against the corresponding longitudinal outcome
Individual-bone scoring or statistical estimation	Individual scoring of RUS, carpal bones, or other features with TW (8, 9); statistical estimation with FELS (1)	Maturity score or bone age	Compares bone groups, versions, and reference standards; prediction requires outcome-specific longitudinal validation
Regional maturity staging	Ossification, morphology, or fusion at the hand, elbow, cervical spine, iliac apophysis, or knee; e.g., Sanders (11), DRU (12), Sauvegrain (15), Risser (10), and CVM (13, 14)	Ordered maturity stage	Locates the current stage of a region; relationships with PHV, local growth, or treatment outcome require longitudinal validation
Regional fusion or bridging assessment	Epiphyseal, physeal, or synchondrosis fusion and MRI-detected bone bridging	Presence, extent, or stage of fusion	Assesses closure in a defined region; validated by longitudinal imaging or actual length change in that region
B. Growth-stage and outcome prediction			
PHV or growth-stage assessment	Serial height velocity or estimation of position relative to PHV using bone age or regional staging	PHV timing or relative stage	Validated against a velocity curve derived from serial height measurements
Adult height or overall remaining-growth prediction	Chronological age, bone age, current height, and other predictors	Predicted adult height or total remaining growth	Follow-up to growth completion with individual prediction error and calibration
Remaining growth of a target segment	Local morphology, atlas, or growth plate status	Predicted local growth, closure time, or risk	Validated against longitudinal length change, closure, or treatment outcome in the target segment
Multi-indicator or multiregional combination	Multiple bone groups, regions, modalities, or clinical features	Joint prediction of a defined outcome	Compared with the best single indicator, with calibration and validation in an independent population

AIS, adolescent idiopathic scoliosis; CVM, cervical vertebral maturation; DRU, distal radius and ulna; FELS, Fels hand-wrist skeletal maturity assessment; GP, Greulich-Pyle; MRI, magnetic resonance imaging; PHV, peak height velocity; RUS, radius ulna and short bones; TW, tanner-whitehouse.

## 4 Direct evidence of discordance between skeletal maturity assessments

The direct human evidence below is organized into Categories I–III according to study design. Category IV mechanistic evidence is considered separately in Section 5.2.

### 4.1 Category I: same-image bone-group comparisons

Comparing bone groups on the same hand-wrist image minimizes differences in examination timing and whole-body developmental state. Carpenter and Lester evaluated 100 pediatric hand-wrist radiographs and found that carpal bone age was 4-25 months younger than the whole-hand assessment on 77 images; overall, the carpal bones and distal radius and ulna also lagged behind the metacarpals and phalanges (3). In TW3-C data from 9,408 healthy children in five Chinese cities, the group mean difference between RUS and carpal bone age was close to zero, yet the standard deviation of individual differences approached one year in some male age groups (20). Longitudinal data further showed different rates and pubertal patterns of score progression between these bone groups (21). Together, these findings show that a near-zero mean bias can coexist with substantial dispersion in paired within-child differences, whereas a high correlation can coexist with a systematic group-level offset. Mean bias, correlation, and the distribution of paired differences should therefore be considered together when individual interpretation is relevant.

Relationships between bone groups within the hand also vary with disease and developmental context. RUS maturation may be more advanced than carpal maturation in children with precocious puberty or congenital adrenal hyperplasia (22). Studies of growth hormone deficiency and hypothyroidism reported differences in the magnitude of delay and in diagnostic discrimination between the two groups (23, 24). In longitudinal Legg-Calve-Perthes disease data, delays in RUS and carpal bone age changed at different rates over the disease course (25). These findings do not support a fixed maturational sequence across clinical conditions. They instead suggest that the direction and meaning of discordance depend on the population, disease stage, comparator, and treatment exposure.

### 4.2 Category II: cross-regional comparisons using different assessment pathways

Paired hand-wrist and knee assessments demonstrate that regional differences within an individual can be much larger than the mean difference in a group. Xi and Roche reported age- and sex-stratified mean absolute differences of approximately 0.34-0.87 years and maximum individual differences of approximately 1.45-2.99 years. The signed mean difference was close to zero, indicating that positive and negative within-child differences could cancel each other at the group level (4). Among 589 referred children, hand-wrist age tended to be lower than knee age in those with delayed maturation, whereas the direction reversed in those with advanced maturation (26). More recently, knee MRI appeared more mature than hand-wrist bone age in 14.1% of adolescents with slowing growth velocity (27). Because several studies changed region, imaging modality, and assessment system simultaneously, these discrepancies cannot be attributed solely to regional biology.

Paired hand-wrist and elbow assessments also diverge, particularly around pubertal onset. Among 211 peripubertal children, the mean absolute deviations from chronological age in boys were 1.26 years for GP hand-wrist assessment and 0.61 years for the Sauvegrain elbow method (28). Another study found that approximately one quarter of paired hand-wrist and elbow estimates differed by more than one year (16). Greater proximity to chronological age in this age window does not establish superior prediction of adult height or segmental remaining growth, because chronological age is not the reference outcome for either question.

### 4.3 Category III: disease- or intervention-based within-person comparisons

Unilateral disease and local treatment provide within-person comparisons under a relatively similar systemic background. In children with Legg-Calve-Perthes disease, hand bone age lagged behind chronological age by approximately 1.4 years, whereas the unaffected hip was slightly advanced; the mean hand-hip difference was approximately 1.7 years (29). Among 78 children with unilateral spastic cerebral palsy, bone age and segmental upper-limb lengths were lower on the affected side than on the unaffected side (all *P* < 0.001). Among the 56 children with hand-function grading, the Manual Ability Classification System (MACS) level was correlated with the side-to-side difference in bone age (r=0.29, *P*=0.03) and with segmental upper-limb length discrepancies (all *P* < 0.05). However, the bone-age difference itself was not significantly correlated with the segmental length discrepancies (30). Follow-up after limb lengthening also demonstrated subsequent growth disturbance and site-specific physeal responses. After subtracting the length gained by distraction, actual lower-limb growth at skeletal maturity was approximately 4.9% lower after tibial lengthening and 8.5% lower after sequential tibial and femoral lengthening than in non-lengthened controls; premature physeal closure occurred predominantly at the proximal tibia and distal femur (31). Unequal residual growth across segments was also observed in congenital lower-limb deformity (32). Within-person designs reduce confounding from genetic, nutritional, and endocrine background, but local disease-related factors such as innervation, loading, perfusion, activity, and treatment remain coupled. Evidence for a local contribution is more persuasive when maturity differences are accompanied by changes in limb length, function, or growth plate behavior. However, these findings cannot serve as regional reference values for healthy children.

### 4.4 Discordance is not universal

Some studies have found strong correlation or substantial categorical agreement. In Italian children, knee bone age assessed by the Roche-Wainer-Thissen (RWT) method was highly correlated with GP or TW hand-wrist bone age (33). Strong associations were also reported among spheno-occipital synchondrosis fusion, hand-wrist maturity, and cervical vertebral maturation, with kappa values of approximately 0.78 for some categorical comparisons (34). Correlation describes whether two indicators change together, whereas paired differences and Bland-Altman limits of agreement describe how far their values differ within the same child (35). Kappa evaluates categorical agreement but is sensitive to marginal category distributions (36). Studies should therefore report group trends, individual paired differences, and stage reclassification to determine whether changing an indicator alters maturity classification or clinical interpretation. Representative direct comparisons are summarized in Table 1.

## 5 Potential sources of discordance

### 5.1 Effects of imaging and assessment methods

Cross-regional comparisons often change several factors simultaneously, including anatomy, imaging modality, observed feature, scoring procedure, and reference standard. Hand-wrist and knee studies have compared FELS or GP assessments with RWT or MRI atlases, whereas hand-wrist and elbow studies have compared GP with the Sauvegrain method (4, 27, 28). Such designs directly establish that two assessment pathways yield different results. They provide stronger evidence about regional effects only when examination timing, side, modality, and assessment rules are controlled as closely as possible.

The assessment method itself can change the output. Bone age is not measured directly from an image; it is an estimate derived by interpreting imaging features according to a specified rule set and reference standard. GP relies on an overall atlas match, TW converts stages assigned to individual bones into scores or ages, and FELS produces a statistical estimate with uncertainty (1). Applying TW2 and TW3 to the same image can change the result (5). Likewise, GP and Sauvegrain assessments can each be reproducible while showing wide paired differences (16). Reproducibility, cross-method agreement, and validity for a clinical outcome are therefore separate properties.

Reference standards are also shaped by birth cohort, geographic setting, and population composition. Hand-wrist data spanning almost a century showed earlier ossification or fusion in later-born cohorts for approximately half of the epiphyses studied, with unequal changes across bone groups (37). Compared with the historical Bolton-Brush sample, a modified FELS knee method suggested advances of 0.74, 0.69, and 0.40 years in modern White boys, Black boys, and Black girls, respectively, whereas an optimized Oxford hip method showed no corresponding shift (38). The direction of temporal differences also varied across shoulder, olecranon, and wrist systems (39). Modern Chinese children may be taller than historical reference populations at the same maturity score (40). These findings describe average differences in specific populations and cannot justify a uniform correction for all contemporary children.

### 5.2 Category IV: mechanistic evidence

Skeletal regions share the same systemic endocrine, nutritional, and growth environment. The growth hormone/insulin-like growth factor 1 axis, thyroid hormone, sex steroids, and glucocorticoids regulate chondrocyte proliferation, hypertrophy, and differentiation and contribute to growth plate senescence and epiphyseal fusion (41, 42). Estrogen accelerates growth plate senescence and earlier exhaustion of proliferative potential (43). However, humans and rabbits undergo pubertal growth plate fusion, whereas commonly used rodents generally do not (42). Animal studies can therefore support biological plausibility but cannot be directly extrapolated to the magnitude or timing of human regional differences. Systemic regulation and local variation are not mutually exclusive. Shared endocrine, nutritional, and genetic influences coordinate overall maturation, while differences in cellular composition, closure sequence, and local environment may produce site-specific responses to the same systemic signals. These findings are consistent with regional differences in developmental timing and response magnitude occurring within a shared systemic regulatory background, rather than implying that skeletal regions develop independently of the rest of the body.

Growth plates at different sites may also differ in prior growth history, cellular reserve, and signal responsiveness. After local glucocorticoid suppression is withdrawn, catch-up growth occurs mainly in the previously suppressed growth plate (44). In hypothyroidism models, reduced growth is accompanied by delayed growth plate senescence, extending the period of growth after thyroid function is restored (45). Age-related declines in insulin-like growth factor 1 receptor expression and proliferation markers also differ among limb growth plates (46). Differences in receptor distribution and leptin response between vertebral and tibial growth plates further show that the same systemic signal can generate site-specific responses (47, 48). These studies do not establish a fixed sequence of regional maturation in humans. Direct evidence in healthy children remains limited because most human studies are cross-sectional imaging comparisons or bilateral assessments in selected diseases.

Local mechanical conditions may further modify growth plate activity. In animal models, changes in tibial physeal loading affect bone length, cell-column number, and tissue architecture (49, 50). Histologic differences have been observed in abnormally loaded regions of AIS vertebral growth plates, and *ex vivo* mechanical stimulation of human growth plate cartilage altered gene expression and signaling pathways (51, 52). The direction of response may depend on load type, magnitude, duration, age, and tissue state. Current evidence therefore supports a possible contribution of mechanics to local developmental variation, not a quantitative relation that can be applied universally in clinical practice.

Taken together, the evidence does not support interpreting every observed discordance as regional biological asynchrony. Category I studies reduce variation in examination timing and imaging modality but retain bone-group-specific assessment effects, whereas Category II studies generally cannot separate anatomy from the assessment pathway. Category III studies support local influences but remain affected by disease and treatment context, and Category IV studies establish mechanistic plausibility without defining the magnitude or sequence of regional differences in healthy children. Observed discordance may therefore arise from assessment methods, regional developmental state, or both, and available studies rarely quantify their relative contributions within an individual child.

When two assessments disagree, interpretation should first clarify what is being compared and whether examination timing, side, imaging modality, method, version, or reference standard differs. Patient and regional context should then be considered before determining whether the discrepancy is large enough to alter a specific growth prediction, risk classification, or treatment decision. Figure 1 summarizes this process.

**Figure 1:**
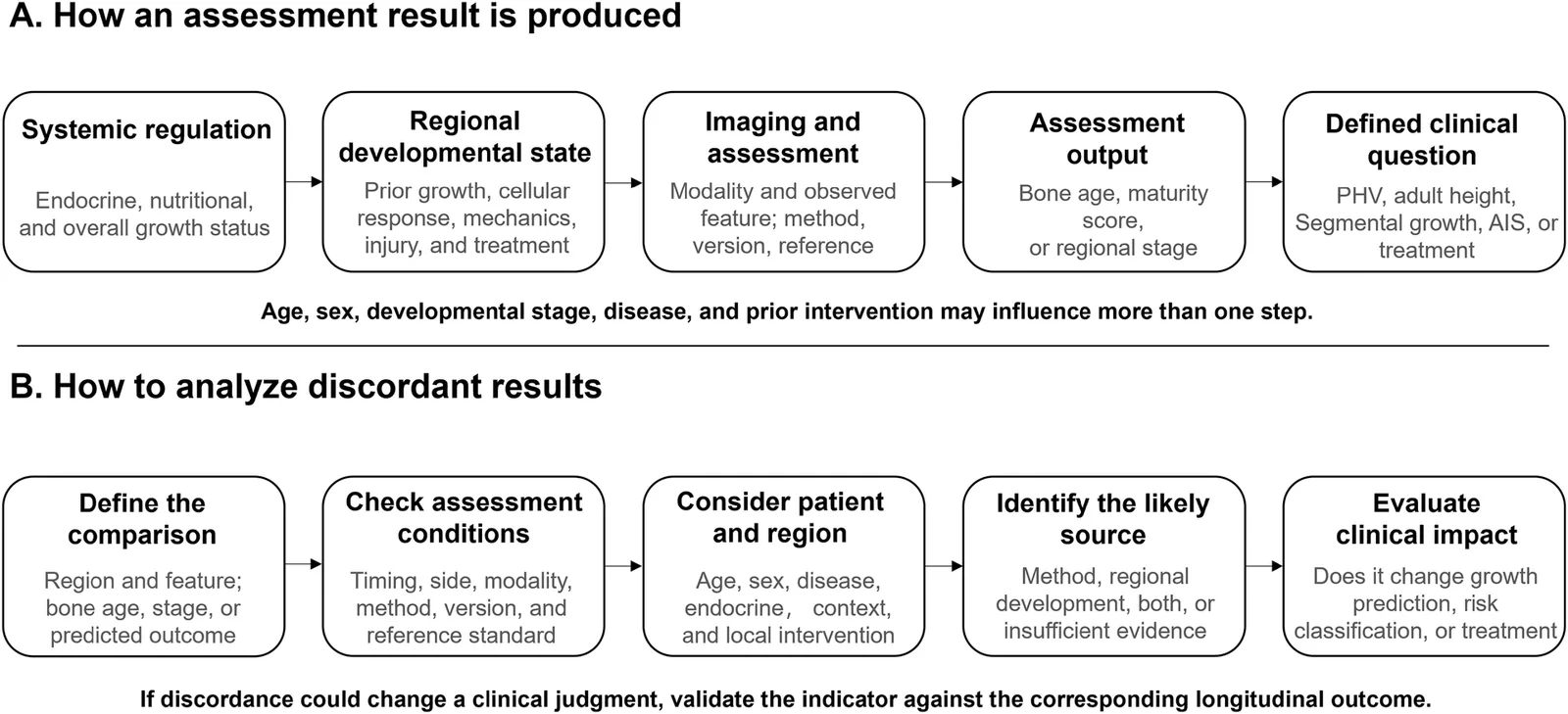
Formation and interpretation of skeletal maturity assessment results. **(A)** Assessment outputs arise from the interaction of systemic regulation, regional developmental state, and the imaging and assessment method before they are applied to a defined clinical question. **(B)** When two results are discordant, the comparison and assessment conditions should be specified first, followed by consideration of patient and regional context, identification of the likely source, and evaluation of whether the difference changes growth prediction, risk classification, or treatment. PHV, peak height velocity; AIS, adolescent idiopathic scoliosis.

## 6 Applicability to specific clinical questions

The clinical value of a skeletal maturity indicator depends on the question it is intended to answer. PHV assessment requires comparison with height-velocity curves derived from serial measurements. Prediction of adult height or remaining growth in a target segment requires follow-up of final height or actual length change in that segment. Brace discontinuation and surgical timing require outcomes such as curve progression, correction achieved, local growth disturbance, and treatment-related complications. The evidence for these distinct questions is considered below.

### 6.1 Identifying peak height velocity

Whether a child is before, near, or after PHV should be established against a velocity curve derived from serial height measurements. In the historical longitudinal cohort reported by Houston (17), RUS bone age was more closely related to PHV timing than carpal bone age. However, individual-level estimates remained imprecise when prediction was attempted more than two years before the cohort's average age at PHV. Calcaneal apophyseal ossification provides relatively fine early staging before PHV, whereas Sanders staging is less discriminating in this window (53). Olecranon ossification, combined with age and sex, can also help estimate proximity to PHV (54). Assessment should therefore focus on the developmental interval covered, the distribution of PHV across stages, and uncertainty in individual timing estimates.

### 6.2 Predicting adult height and overall remaining growth

Adult-height models should report mean bias, absolute error, and agreement between predicted and observed values. A mean bias near zero may reflect cancellation of overestimation and underestimation; the distribution of individual error remains essential, and Bland-Altman analysis can display that distribution (35). Prediction studies should clearly report internal validation, calibration, and, where available, external validation (55). When a new model is proposed as an addition to an existing assessment, its incremental performance should also be compared with an appropriate baseline model.

Longitudinal data from healthy children suggest that chronological age-based multiplier models perform well before the growth spurt, whereas PHV-timing models are more accurate for height and limb-length prediction after the spurt begins (56). In 173 postmenarchal girls, a model combining height at menarche, GP hand-wrist bone age, and distal femoral radiomic features outperformed traditional methods. The fitted model had a coefficient of determination of 0.733, and the cross-validated mean bias was near zero, but the 95% limits of agreement remained approximately ± 4 cm (57). Multiregional information may therefore be complementary, but individual prediction error and transportability remain uncertain.

### 6.3 Predicting remaining growth in a target skeletal segment

When the clinical question concerns a specific skeletal segment, local morphology and growth plate status may be closer to the target outcome. A model based on proximal tibial metaphyseal width and lateral epiphyseal height explained approximately 76% of the variation in remaining growth in a held-out sample from the same institution (18). An ankle atlas also provided estimates of late remaining distal tibial and fibular growth (58). Local indicators are not necessarily superior to a distally measured indicator that has been validated longitudinally: for growth around the knee, automated GP hand-wrist bone age produced smaller mean absolute errors for the femur and tibia than chronological age or Sauvegrain staging (2). Local imaging, established hand-wrist assessment, and their combination should therefore be compared in the same population against actual longitudinal growth of the target segment.

### 6.4 AIS and other orthopedic treatment decisions

AIS encompasses several questions, including growth stage, curve progression, brace discontinuation, and timing of growth-modulation procedures, and therefore illustrates why indicator performance is task dependent. A systematic review and meta-analysis reported a pooled mature-vs.-immature discrepancy rate of 16.1% between the Risser sign and Sanders staging (59). Longitudinal data further showed that a single Risser category can encompass several Sanders stages and that the Risser sign, Sanders staging, and the distal radius and ulna (DRU) classification provide different information about height velocity, curve progression, and growth plateau (60). Among a small subgroup with discordant hand staging, observed height velocity could also move in the opposite direction from the stage suggested by the Risser sign (61). Longitudinal comparison of the thumb ossification composite index (TOCI) and Sanders staging likewise showed different concentrations of PHV across their respective stages (62). These findings support selecting an indicator for a defined outcome rather than ranking systems by overall cross-sectional association.

Brace-discontinuation studies connect maturity indicators more directly to curve outcomes. In a scoping review, 236 of 700 patients (34%) across seven studies that reported progression greater than 5 degrees experienced progression two to four years after brace cessation or weaning (63). In a prospective cohort of 144 patients, bracing was stopped at Risser stage 4, at least two years after menarche, and after six months without height increase. Even under these conventional criteria, 29% progressed by more than 5 degrees, whereas later Sanders stages and DRU grades were associated with lower risk (64). These percentages arose from a heterogeneous subset of a review and from a single-center protocol, respectively, and should not be interpreted as a universal incidence or universal threshold for brace discontinuation.

Vertebral body tethering (VBT) concerns overall height, leg-length growth, and spinal curve modulation. In a 34-patient cohort followed to maturity, TOCI and Sanders staging performed relatively well in models of leg-length growth, whereas cervical vertebral maturation and TOCI were more closely related to curve modulation (65). The small, selected surgical sample limits generalization of the fitted models. Other orthopedic interventions target different outcomes: anterior cruciate ligament reconstruction emphasizes local physeal status, epiphysiodesis emphasizes growth remaining for correction, and limb lengthening emphasizes actual postintervention growth loss (31, 66, 67). Indicators should be validated separately against the relevant skeletal segment and observed treatment outcome.

### 6.5 Combining multiple indicators

One rationale for combining indicators is to extend the developmental interval that can be observed. Once olecranon staging reaches later phases, Sanders staging and the DRU classification can continue to distinguish subsequent maturation (68). Calcaneal and hand indicators likewise provide information in different intervals before and after PHV (53). In this setting, independent outputs can locate the child within complementary staging windows without first forcing all indicators into a single age estimate.

A second rationale is to improve prediction of a defined outcome. Combined hand-wrist and knee imaging models and multiregional ultrasound models suggest that regional information may be complementary (57, 69). Prediction-model performance should be reported with calibration and, where available, external validation (55). The incremental value of combining indicators should be tested against an appropriate well-performing single-indicator model, with individual-level error also reported. A second region or indicator has practical value only if it reduces uncertainty or changes a clinical judgment. Existing clinical images should be used whenever possible to avoid additional radiation or examination burden solely for model development.

Non-radiographic variables may provide complementary information because they can capture aspects of current pubertal or growth-related biological activity that are not fully represented by radiographic morphology. In boys, serum testosterone and estradiol have been associated with growth velocity approaching PHV (70). In a large cross-sectional cohort, estradiol, luteinizing hormone, follicle-stimulating hormone, and testosterone were also associated with bone age (71). A systematic review identified stage-dependent changes in IGF-1 and several bone-turnover biomarkers, although methodological heterogeneity limited quantitative synthesis and warrants caution in individual prediction (72). In a prospective multicenter cohort of 254 patients with idiopathic scoliosis, collagen X biomarker (CXM) explained 50% of the variance in height velocity. Combining CXM with the Risser sign increased the explained variance to 55% (73). These findings support evaluating whether non-radiographic measures add useful information beyond established clinical and imaging indicators when predicting defined longitudinal growth outcomes. Table 2 summarizes representative outcome-linked evidence and its principal limitations.

## 7 Discussion and research priorities

### 7.1 Separating regional and methodological effects

The central interpretive problem is that an observed difference may arise from the assessment pathway, regional development, or both. Future paired studies should separately compare methods within the same region and regions within the same child under contemporaneous, same-side, and otherwise comparable conditions. Linking these comparisons to longitudinal growth outcomes would help distinguish method-related from regional contributions. Studies should report individual paired differences and stage reclassification rather than relying only on correlations or group means.

### 7.2 Building contemporaneous multiregional longitudinal cohorts

Multiregional cohorts should record regional imaging at several time points and link it to PHV, adult height, target-segment growth, AIS curve progression, or treatment response. Existing multiregional MRI data describe population-level closure sequences and their relationships with sex, pubertal status, and body mass index (74). The next step is to estimate individual trajectories and determine how regional differences vary with age and disease context.

Repeated pediatric imaging should preferentially use examinations obtained during clinical care and, where appropriate, MRI or ultrasound rather than additional radiographs collected only to assemble multiregional data. Unilateral disease, local injury, and local treatment can function as natural experiments, as shown in Legg-Calve-Perthes disease and unilateral spastic cerebral palsy (29, 30). Such designs can help evaluate local influences but cannot establish normative reference values for regional maturation in healthy populations. Prediction models also require external validation across centers, devices, and populations.

### 7.3 Using artificial intelligence to predict longitudinal growth outcomes

Artificial intelligence should not be limited to reproducing a conventional manually assigned bone age. Future models should compare region-specific imaging information with clinical, anthropometric, hormonal, and biomarker data to determine whether multimodal combinations improve prediction of defined longitudinal growth outcomes. External validation should assess calibration and individual-level prediction error (55), followed by evaluation of whether improved prediction meaningfully changes clinical decisions. The major evidence gaps and corresponding study designs are summarized in Table 4.

**Table 4:** Major evidence gaps and corresponding research designs.

Major evidence gap	Recommended design	Principal outcomes and analyses	Contribution and limitation
Cross-regional comparisons change both region and method	Contemporaneous, same-side, within-person regional comparisons with modality and observation rules aligned when possible	Signed and absolute paired differences, distributions, and stage reclassification	Estimates regional differences under more comparable conditions; does not establish mechanism alone
Methods or versions differ within the same region	Apply methods, versions, or algorithms to the same image	Observer/algorithm error, paired differences, and Bland-Altman analysis	Estimates method-related effects; does not address regional biology
Few multiregional longitudinal data within the same child	Longitudinal cohorts with repeated multiregional assessment linked to observed outcomes	PHV, adult height, target-segment growth, or treatment response	Describes individual trajectories; cannot establish universal thresholds without external validation
Limited evidence on local influences	Unilateral disease, local injury, or treatment with contralateral or appropriate controls	Local and systemic factors, temporal sequence, and repeated measures	Can evaluate local influences; cannot establish normative reference values for healthy populations or identify a unique mechanism
Uncertain applicability of historical standards	Modern multicenter validation stratified by cohort, geography, sex, and population	Systematic bias, age/sex strata, and longitudinal outcomes	Identifies population shifts; does not establish predictive validity by itself
Combined models may duplicate information or overfit, and the added value of non-radiographic features remains uncertain	Compare prespecified clinical and anthropometric, imaging-only, biomarker-only, and combined models; perform multicenter external validation	Individual error, calibration, discrimination where applicable, external performance, and decision impact	Tests incremental value; hormone and biomarker effects may vary by sex, developmental stage, disease context, and assay

### 7.4 Limitations

This review was limited by methodological heterogeneity and the uneven distribution of evidence across skeletal regions, populations, imaging modalities, and clinical outcomes, which precluded quantitative synthesis and restricted direct comparisons between studies. Because formal design-specific risk-of-bias tools were not applied, the review emphasizes study design, comparability, applicability, and consistency of findings rather than pooled estimates of comparative performance.

## 8 Conclusions

Different skeletal maturity indicators can yield discordant age estimates or stage classifications in the same child. Sources include imaging and scoring methods, method versions, reference standards, and regional developmental state. Such discrepancies do not by themselves demonstrate regional biological asynchrony because anatomical region, imaging modality, scoring method, and reference standard often change together. Established bone age methods and maturity stages remain clinically valuable, but they should be selected and validated against the intended outcome, whether PHV, adult height, target-segment growth, or a treatment-specific endpoint. Clinical interpretation should therefore consider the distribution of individual-level disagreement and the frequency of stage reclassification. Contemporaneous paired designs, multiregional longitudinal follow-up, and multicenter external validation are needed to clarify how discordance should be interpreted and to determine whether its magnitude is sufficient to alter clinical judgment.
