# Supplementary material for: Discordance among skeletal maturity indicators: sources, interpretation, and clinical implications
Source: Front Pediatr. 2026 Sep 23;14:1970006. doi: 10.3389/fped.2026.1970006 (PMC13643382; doi:10.3389/fped.2026.1970006)
Supplement: Supplementary file 1 [file Datasheet1.docx]

**Supplementary Table S1. Complete database search strategies**

| **Database** | **Module and evidence domain** | **Search field** | **Search date** | **Database filters applied** | **Records retrieved** | **Complete search string** |
| --- | --- | --- | --- | --- | --- | --- |
| **PubMed** | G1: Cross-region or cross-indicator discordance Direct comparisons between skeletal regions or maturity indicators within the same individual | MeSH terms and title/abstract fields | 27 July 2026 | None | 541 | ("Age Determination by Skeleton"[Mesh] OR "bone age"[tiab] OR "skeletal age"[tiab] OR "skeletal maturation"[tiab] OR "skeletal maturity"[tiab] OR "bone maturation"[tiab] OR "skeletal maturity index"[tiab] OR "skeletal maturity indices"[tiab] OR "growth plate maturation"[tiab] OR "physeal maturation"[tiab] OR "epiphyseal maturation"[tiab] OR "epiphyseal fusion"[tiab] OR "physeal fusion"[tiab]) AND (discordan*[tiab] OR asynchron*[tiab] OR heterochron*[tiab] OR multisite[tiab] OR "multi-site"[tiab] OR "multiple sites"[tiab] OR "multiple regions"[tiab] OR "axial and appendicular"[tiab] OR "axial versus appendicular"[tiab] OR ((hand[tiab] OR wrist[tiab]) AND (elbow[tiab] OR olecranon[tiab] OR shoulder[tiab] OR pelvis[tiab] OR hip[tiab] OR spine[tiab] OR vertebr*[tiab] OR knee[tiab] OR ankle[tiab] OR foot[tiab] OR calcane*[tiab] OR clavicl*[tiab])) OR (knee[tiab] AND (ankle[tiab] OR wrist[tiab]))) |
| **PubMed** | G2: Within-hand RUS–carpal differences Differences between carpal and radius–ulna–short-bone maturation or other hand compartments | MeSH terms and title/abstract fields | 27 July 2026 | None | 142 | ("Age Determination by Skeleton"[Mesh] OR "bone age"[tiab] OR "skeletal age"[tiab] OR "skeletal maturation"[tiab] OR "skeletal maturity"[tiab] OR "bone maturation"[tiab] OR "skeletal maturity index"[tiab] OR "skeletal maturity indices"[tiab] OR "growth plate maturation"[tiab] OR "physeal maturation"[tiab] OR "epiphyseal maturation"[tiab] OR "epiphyseal fusion"[tiab] OR "physeal fusion"[tiab]) AND (((RUS[tiab] OR "radius ulna short bones"[tiab] OR "radius ulna and short bones"[tiab] OR "tubular bones"[tiab] OR phalang*[tiab] OR metacarp*[tiab]) AND (carpal*[tiab] OR wrist[tiab])) OR "segmental bone age"[tiab] OR "segmental skeletal maturity"[tiab]) AND (discord*[tiab] OR differen*[tiab] OR compar*[tiab] OR segmental[tiab] OR regional[tiab] OR compartment*[tiab] OR advanc*[tiab] OR delay*[tiab]) |
| **PubMed** | G3: Regional timing and growth-plate mechanisms Regional developmental timing, physeal closure sequences, and site-specific growth-plate mechanisms | MeSH terms and title/abstract fields | 27 July 2026 | None | 558 | ((("Growth Plate"[Mesh] OR "epiphyseal fusion"[tiab] OR "physeal fusion"[tiab] OR "bone age"[tiab] OR "skeletal age"[tiab] OR "skeletal maturation"[tiab] OR "skeletal maturity"[tiab]) AND (regional[tiab] OR "site-specific"[tiab] OR "site specific"[tiab] OR segmental[tiab] OR multisite[tiab] OR "multiple sites"[tiab] OR "fusion sequence"[tiab] OR "closure sequence"[tiab] OR "axial and appendicular"[tiab] OR "upper limb"[tiab] OR "lower limb"[tiab] OR "upper extremity"[tiab] OR "lower extremity"[tiab]) AND (timing[tiab] OR sequence*[tiab] OR tempo[tiab] OR closure[tiab] OR fusion[tiab] OR "peak height velocity"[tiab] OR senescence[tiab] OR mechanobiolog*[tiab] OR "mechanical loading"[tiab] OR "local regulation"[tiab])) OR (("Growth Plate"[Mesh] OR "growth plate"[tiab] OR physeal[tiab]) AND (matur*[tiab] OR senescence[tiab] OR fusion[tiab] OR closure[tiab]) AND (endocrine[tiab] OR hormone*[tiab] OR mechanobiolog*[tiab] OR "mechanical loading"[tiab] OR "local regulation"[tiab] OR paracrine[tiab] OR PTHrP[tiab] OR hedgehog[tiab] OR BMP[tiab] OR FGF[tiab] OR Wnt[tiab]))) |
| **PubMed** | G4: Task-specific validity Validation against peak height velocity, final height, remaining growth, local growth, or clinical outcomes | MeSH terms and title/abstract fields | 27 July 2026 | None | 1,003 | ("Age Determination by Skeleton"[Mesh] OR "bone age"[tiab] OR "skeletal age"[tiab] OR "skeletal maturation"[tiab] OR "skeletal maturity"[tiab] OR "bone maturation"[tiab] OR "skeletal maturity index"[tiab] OR "skeletal maturity indices"[tiab] OR "growth plate maturation"[tiab] OR "physeal maturation"[tiab] OR "epiphyseal maturation"[tiab] OR "epiphyseal fusion"[tiab] OR "physeal fusion"[tiab]) AND ("peak height velocity"[tiab] OR "adult height"[tiab] OR "final height"[tiab] OR "height prediction"[tiab] OR "remaining growth"[tiab] OR "growth remaining"[tiab] OR "residual growth"[tiab] OR "growth potential"[tiab] OR "leg length"[tiab] OR "limb length"[tiab] OR "local growth"[tiab] OR "longitudinal growth"[tiab] OR "age threshold"[tiab] OR "chronological age estimation"[tiab]) AND (predict*[tiab] OR valid*[tiab] OR accur*[tiab] OR "predictive value"[tiab] OR "prediction error"[tiab]) |
| **PubMed** | G5: Disease-, environment-, or intervention-related regional differences Regional differences associated with disease, endocrine status, nutrition, injury, loading, or intervention | MeSH terms and title/abstract fields | 27 July 2026 | None | 132 | ("Age Determination by Skeleton"[Mesh] OR "bone age"[tiab] OR "skeletal age"[tiab] OR "skeletal maturation"[tiab] OR "skeletal maturity"[tiab] OR "bone maturation"[tiab] OR "skeletal maturity index"[tiab] OR "skeletal maturity indices"[tiab] OR "growth plate maturation"[tiab] OR "physeal maturation"[tiab] OR "epiphyseal maturation"[tiab] OR "epiphyseal fusion"[tiab] OR "physeal fusion"[tiab]) AND (discord*[tiab] OR asynchron*[tiab] OR segmental[tiab] OR compartment*[tiab] OR ((RUS[tiab] OR carpal*[tiab] OR "tubular bones"[tiab]) AND (differen*[tiab] OR compar*[tiab] OR advanc*[tiab] OR delay*[tiab])) OR "local growth plate"[tiab] OR "growth plate injury"[tiab] OR "physeal injury"[tiab]) AND ("Growth Disorders"[Mesh] OR "Obesity"[Mesh] OR "Hypothyroidism"[Mesh] OR "Adrenal Hyperplasia, Congenital"[Mesh] OR "Puberty, Precocious"[Mesh] OR "Kidney Diseases"[Mesh] OR "Glucocorticoids"[Mesh] OR obes*[tiab] OR hypothyroid*[tiab] OR "precocious puberty"[tiab] OR "congenital adrenal hyperplasia"[tiab] OR "growth hormone deficien*"[tiab] OR renal[tiab] OR "chronic kidney disease"[tiab] OR glucocorticoid*[tiab] OR malnutrition[tiab] OR "skeletal dysplasia*"[tiab] OR injur*[tiab] OR trauma*[tiab] OR "mechanical loading"[tiab]) |
| **PubMed** | G6: AI, regional, and opportunistic imaging Regional, multiregional, interpretable, or opportunistic imaging approaches, including artificial intelligence | MeSH terms and title/abstract fields | 27 July 2026 | None | 146 | ("Age Determination by Skeleton"[Mesh] OR "bone age"[tiab] OR "skeletal age"[tiab] OR "skeletal maturation"[tiab] OR "skeletal maturity"[tiab] OR "bone maturation"[tiab] OR "skeletal maturity index"[tiab] OR "skeletal maturity indices"[tiab] OR "growth plate maturation"[tiab] OR "physeal maturation"[tiab] OR "epiphyseal maturation"[tiab] OR "epiphyseal fusion"[tiab] OR "physeal fusion"[tiab]) AND ("Artificial Intelligence"[Mesh] OR "Machine Learning"[Mesh] OR "Deep Learning"[Mesh] OR "artificial intelligence"[tiab] OR "machine learning"[tiab] OR "deep learning"[tiab] OR "neural network*"[tiab] OR automated[tiab] OR automation[tiab]) AND (segmental[tiab] OR regional[tiab] OR "multi-region"[tiab] OR multiregion[tiab] OR "region of interest"[tiab] OR ROI[tiab] OR local*[tiab] OR anatom*[tiab] OR interpretab*[tiab] OR explainab*[tiab] OR opportunistic[tiab] OR elbow[tiab] OR olecranon[tiab] OR knee[tiab] OR ankle[tiab] OR MRI[tiab]) |
| **Scopus** | G1: Cross-region or cross-indicator discordance Direct comparisons between skeletal regions or maturity indicators within the same individual | TITLE-ABS-KEY | 27 July 2026 | None | 1,010 | TITLE-ABS-KEY(("bone age" OR "skeletal age" OR "skeletal maturation" OR "skeletal maturity" OR "bone maturation" OR "skeletal maturity index" OR "skeletal maturity indices" OR "growth plate maturation" OR "physeal maturation" OR "epiphyseal maturation" OR "epiphyseal fusion" OR "physeal fusion") AND (discordan* OR asynchron* OR heterochron* OR multisite OR "multi-site" OR "multiple sites" OR "multiple regions" OR "axial and appendicular" OR "axial versus appendicular" OR ((hand OR wrist) AND (elbow OR olecranon OR shoulder OR pelvis OR hip OR spine OR vertebr* OR knee OR ankle OR foot OR calcane* OR clavicl*)) OR (knee AND (ankle OR wrist)))) |
| **Scopus** | G2: Within-hand RUS–carpal differences Differences between carpal and radius–ulna–short-bone maturation or other hand compartments | TITLE-ABS-KEY | 27 July 2026 | None | 258 | TITLE-ABS-KEY(("bone age" OR "skeletal age" OR "skeletal maturation" OR "skeletal maturity" OR "bone maturation" OR "skeletal maturity index" OR "skeletal maturity indices" OR "growth plate maturation" OR "physeal maturation" OR "epiphyseal maturation" OR "epiphyseal fusion" OR "physeal fusion") AND (((RUS OR "radius ulna short bones" OR "radius ulna and short bones" OR "tubular bones" OR phalang* OR metacarp*) AND (carpal* OR wrist)) OR "segmental bone age" OR "segmental skeletal maturity") AND (discord* OR differen* OR compar* OR segmental OR regional OR compartment* OR advanc* OR delay*)) |
| **Scopus** | G3: Regional timing and growth-plate mechanisms Regional developmental timing, physeal closure sequences, and site-specific growth-plate mechanisms | TITLE-ABS-KEY | 27 July 2026 | None | 958 | TITLE-ABS-KEY((("growth plate" OR "epiphyseal fusion" OR "physeal fusion" OR "bone age" OR "skeletal age" OR "skeletal maturation" OR "skeletal maturity") AND (regional OR "site-specific" OR "site specific" OR segmental OR multisite OR "multiple sites" OR "fusion sequence" OR "closure sequence" OR "axial and appendicular" OR "upper limb" OR "lower limb" OR "upper extremity" OR "lower extremity") AND (timing OR sequence* OR tempo OR closure OR fusion OR "peak height velocity" OR senescence OR mechanobiolog* OR "mechanical loading" OR "local regulation")) OR (("growth plate" OR physeal) AND (matur* OR senescence OR fusion OR closure) AND (endocrine OR hormone* OR mechanobiolog* OR "mechanical loading" OR "local regulation" OR paracrine OR PTHrP OR hedgehog OR BMP OR FGF OR Wnt))) |
| **Scopus** | G4: Task-specific validity Validation against peak height velocity, final height, remaining growth, local growth, or clinical outcomes | TITLE-ABS-KEY | 27 July 2026 | None | 1,266 | TITLE-ABS-KEY(("bone age" OR "skeletal age" OR "skeletal maturation" OR "skeletal maturity" OR "bone maturation" OR "skeletal maturity index" OR "skeletal maturity indices" OR "growth plate maturation" OR "physeal maturation" OR "epiphyseal maturation" OR "epiphyseal fusion" OR "physeal fusion") AND ("peak height velocity" OR "adult height" OR "final height" OR "height prediction" OR "remaining growth" OR "growth remaining" OR "residual growth" OR "growth potential" OR "leg length" OR "limb length" OR "local growth" OR "longitudinal growth" OR "age threshold" OR "chronological age estimation") AND (predict* OR valid* OR accur* OR "predictive value" OR "prediction error")) |
| **Scopus** | G5: Disease-, environment-, or intervention-related regional differences Regional differences associated with disease, endocrine status, nutrition, injury, loading, or intervention | TITLE-ABS-KEY | 27 July 2026 | None | 290 | TITLE-ABS-KEY(("bone age" OR "skeletal age" OR "skeletal maturation" OR "skeletal maturity" OR "bone maturation" OR "skeletal maturity index" OR "skeletal maturity indices" OR "growth plate maturation" OR "physeal maturation" OR "epiphyseal maturation" OR "epiphyseal fusion" OR "physeal fusion") AND (discord* OR asynchron* OR segmental OR compartment* OR ((RUS OR carpal* OR "tubular bones") AND (differen* OR compar* OR advanc* OR delay*)) OR "local growth plate" OR "growth plate injury" OR "physeal injury") AND ("growth disorder*" OR obes* OR hypothyroid* OR "precocious puberty" OR "congenital adrenal hyperplasia" OR "growth hormone deficien*" OR renal OR "chronic kidney disease" OR glucocorticoid* OR malnutrition OR "skeletal dysplasia*" OR injur* OR trauma* OR "mechanical loading")) |
| **Scopus** | G6: AI, regional, and opportunistic imaging Regional, multiregional, interpretable, or opportunistic imaging approaches, including artificial intelligence | TITLE-ABS-KEY | 27 July 2026 | None | 372 | TITLE-ABS-KEY(("bone age" OR "skeletal age" OR "skeletal maturation" OR "skeletal maturity" OR "bone maturation" OR "skeletal maturity index" OR "skeletal maturity indices" OR "growth plate maturation" OR "physeal maturation" OR "epiphyseal maturation" OR "epiphyseal fusion" OR "physeal fusion") AND ("artificial intelligence" OR "machine learning" OR "deep learning" OR "neural network*" OR automated OR automation) AND (segmental OR regional OR "multi-region" OR multiregion OR "region of interest" OR ROI OR local* OR anatom* OR interpretab* OR explainab* OR opportunistic OR elbow OR olecranon OR knee OR ankle OR MRI)) |
| **Web of Science Core Collection** | G1: Cross-region or cross-indicator discordance Direct comparisons between skeletal regions or maturity indicators within the same individual | Topic field (TS) | 27 July 2026 | None | 474 | TS=(("bone age" OR "skeletal age" OR "skeletal maturation" OR "skeletal maturity" OR "bone maturation" OR "skeletal maturity index" OR "skeletal maturity indices" OR "growth plate maturation" OR "physeal maturation" OR "epiphyseal maturation" OR "epiphyseal fusion" OR "physeal fusion") AND (discordan* OR asynchron* OR heterochron* OR multisite OR "multi-site" OR "multiple sites" OR "multiple regions" OR "axial and appendicular" OR "axial versus appendicular" OR ((hand OR wrist) AND (elbow OR olecranon OR shoulder OR pelvis OR hip OR spine OR vertebr* OR knee OR ankle OR foot OR calcane* OR clavicl*)) OR (knee AND (ankle OR wrist)))) |
| **Web of Science Core Collection** | G2: Within-hand RUS–carpal differences Differences between carpal and radius–ulna–short-bone maturation or other hand compartments | Topic field (TS) | 27 July 2026 | None | 83 | TS=(("bone age" OR "skeletal age" OR "skeletal maturation" OR "skeletal maturity" OR "bone maturation" OR "skeletal maturity index" OR "skeletal maturity indices" OR "growth plate maturation" OR "physeal maturation" OR "epiphyseal maturation" OR "epiphyseal fusion" OR "physeal fusion") AND (((RUS OR "radius ulna short bones" OR "radius ulna and short bones" OR "tubular bones" OR phalang* OR metacarp*) AND (carpal* OR wrist)) OR "segmental bone age" OR "segmental skeletal maturity") AND (discord* OR differen* OR compar* OR segmental OR regional OR compartment* OR advanc* OR delay*)) |
| **Web of Science Core Collection** | G3: Regional timing and growth-plate mechanisms Regional developmental timing, physeal closure sequences, and site-specific growth-plate mechanisms | Topic field (TS) | 27 July 2026 | None | 638 | TS=((("growth plate" OR "epiphyseal fusion" OR "physeal fusion" OR "bone age" OR "skeletal age" OR "skeletal maturation" OR "skeletal maturity") AND (regional OR "site-specific" OR "site specific" OR segmental OR multisite OR "multiple sites" OR "fusion sequence" OR "closure sequence" OR "axial and appendicular" OR "upper limb" OR "lower limb" OR "upper extremity" OR "lower extremity") AND (timing OR sequence* OR tempo OR closure OR fusion OR "peak height velocity" OR senescence OR mechanobiolog* OR "mechanical loading" OR "local regulation")) OR (("growth plate" OR physeal) AND (matur* OR senescence OR fusion OR closure) AND (endocrine OR hormone* OR mechanobiolog* OR "mechanical loading" OR "local regulation" OR paracrine OR PTHrP OR hedgehog OR BMP OR FGF OR Wnt))) |
| **Web of Science Core Collection** | G4: Task-specific validity Validation against peak height velocity, final height, remaining growth, local growth, or clinical outcomes | Topic field (TS) | 27 July 2026 | None | 652 | TS=(("bone age" OR "skeletal age" OR "skeletal maturation" OR "skeletal maturity" OR "bone maturation" OR "skeletal maturity index" OR "skeletal maturity indices" OR "growth plate maturation" OR "physeal maturation" OR "epiphyseal maturation" OR "epiphyseal fusion" OR "physeal fusion") AND ("peak height velocity" OR "adult height" OR "final height" OR "height prediction" OR "remaining growth" OR "growth remaining" OR "residual growth" OR "growth potential" OR "leg length" OR "limb length" OR "local growth" OR "longitudinal growth" OR "age threshold" OR "chronological age estimation") AND (predict* OR valid* OR accur* OR "predictive value" OR "prediction error")) |
| **Web of Science Core Collection** | G5: Disease-, environment-, or intervention-related regional differences Regional differences associated with disease, endocrine status, nutrition, injury, loading, or intervention | Topic field (TS) | 27 July 2026 | None | 84 | TS=(("bone age" OR "skeletal age" OR "skeletal maturation" OR "skeletal maturity" OR "bone maturation" OR "skeletal maturity index" OR "skeletal maturity indices" OR "growth plate maturation" OR "physeal maturation" OR "epiphyseal maturation" OR "epiphyseal fusion" OR "physeal fusion") AND (discord* OR asynchron* OR segmental OR compartment* OR ((RUS OR carpal* OR "tubular bones") AND (differen* OR compar* OR advanc* OR delay*)) OR "local growth plate" OR "growth plate injury" OR "physeal injury") AND ("growth disorder*" OR obes* OR hypothyroid* OR "precocious puberty" OR "congenital adrenal hyperplasia" OR "growth hormone deficien*" OR renal OR "chronic kidney disease" OR glucocorticoid* OR malnutrition OR "skeletal dysplasia*" OR injur* OR trauma* OR "mechanical loading")) |
| **Web of Science Core Collection** | G6: AI, regional, and opportunistic imaging Regional, multiregional, interpretable, or opportunistic imaging approaches, including artificial intelligence | Topic field (TS) | 27 July 2026 | None | 145 | TS=(("bone age" OR "skeletal age" OR "skeletal maturation" OR "skeletal maturity" OR "bone maturation" OR "skeletal maturity index" OR "skeletal maturity indices" OR "growth plate maturation" OR "physeal maturation" OR "epiphyseal maturation" OR "epiphyseal fusion" OR "physeal fusion") AND ("artificial intelligence" OR "machine learning" OR "deep learning" OR "neural network*" OR automated OR automation) AND (segmental OR regional OR "multi-region" OR multiregion OR "region of interest" OR ROI OR local* OR anatom* OR interpretab* OR explainab* OR opportunistic OR elbow OR olecranon OR knee OR ankle OR MRI)) |

Note: The reported yields are raw module-level results before deduplication. Records were subsequently deduplicated across both databases and search modules. No database filters were applied for publication year, language, study design, population, or publication type. Grey literature was not systematically searched.

**Supplementary Table S2. Eligibility criteria for evidence selection**

| **Domain** | **Inclusion criteria** | **Exclusion criteria or handling** |
| --- | --- | --- |
| **Core comparison** | Studies comparing two or more skeletal regions, bone groups, maturity indicators, scoring systems, or method versions within the same or a clinically comparable population. | Studies assessing only one maturity method without a regional, cross-method, or outcome-linked comparison. |
| **Regional development** | Studies characterizing regional ossification, physeal closure, fusion sequence, growth velocity, or maturation timing. | Purely descriptive anatomical reports without interpretable relevance to skeletal maturation or regional timing. |
| **Outcome-linked validity** | Studies evaluating an indicator against peak height velocity, final height, remaining global or segmental growth, scoliosis behavior, treatment response, or another defined clinical endpoint. | Studies reporting only correlation with chronological age, reliability, or technical accuracy without a relevant comparison or longitudinal/clinical target. |
| **Populations** | Healthy children and adolescents; adolescents with idiopathic scoliosis; and populations with endocrine, developmental, neuromuscular, skeletal, or other growth-related conditions when they informed regional discordance or external validity. | Adult-only populations without information relevant to skeletal maturation; isolated rare cases without an incremental conceptual or mechanistic contribution. |
| **Human study designs** | Cross-sectional comparisons, longitudinal cohorts, diagnostic or prognostic studies, clinical outcome studies, and studies of disease or local intervention. | Treatment studies in which skeletal maturity was only a baseline descriptor and was not related to comparison, growth, prognosis, or treatment outcome. |
| **Mechanistic evidence** | Animal, laboratory, or human-tissue studies providing site-specific evidence on growth-plate maturation, senescence, endocrine response, mechanical loading, or local regulation. | Non-human or laboratory studies addressing only general molecular pathways without site-specific or regional maturation relevance. |
| **Artificial intelligence and imaging** | Studies providing regional, multiregional, interpretable, or outcome-linked maturity information from radiography, MRI, ultrasound, or artificial intelligence. | Algorithm studies reporting only overall age-prediction performance without regional, cross-method, or outcome-linked information. |
| **Methodological and contextual sources** | Primary publications describing established assessment systems, authoritative methodological papers, relevant reviews, and clinical guidance documents used to define methods, provide context, or support evidence navigation. | Commentary or background material without a necessary methodological, interpretive, or clinical-context role. |
| **Forensic literature** | Forensic studies were eligible only when they provided transferable evidence on regional maturation, fusion sequence, population calibration, or methodological comparability. | Studies focused exclusively on chronological-age estimation for forensic classification without relevance to the review framework. |
| **Publication year, language, and publication type** | No database filters were applied for publication year, language, study design, population, or publication type. | Grey literature was not systematically searched. Reports retrieved from the selected databases were not excluded solely because of publication year or language. |

**Supplementary Table S3. Additional eligible sources retained through targeted supplementary searches, source verification, and citation tracking**

| **Reference** | **Route of identification** | **Role in the review** |
| --- | --- | --- |
| 7. Greulich and Pyle, 1959 | Primary-method source verification during revision | Authoritative source for the Greulich-Pyle atlas |
| 8. Tanner et al., 1983 | Primary-method source verification during revision | Authoritative source for the TW2 method |
| 9. Tanner et al., 2001 | Primary-method source verification during revision | Authoritative source for the TW3 method |
| 10. Risser, 1958 | Primary-method source verification during revision | Primary description of the Risser sign |
| 11. Sanders et al., 2008 | Primary-method source verification during revision | Primary methodological source for simplified digital maturity staging |
| 12. Luk et al., 2014 | Primary-method source verification during revision | Primary methodological source for the DRU classification |
| 13. Hassel and Farman, 1995 | Primary-method source verification during revision | Foundational source for cervical vertebral maturation assessment |
| 14. Baccetti et al., 2002 | Primary-method source verification during revision | Methodological refinement of cervical vertebral maturation assessment |
| 70. Albin and Norjavaara, 2013 | Targeted supplementary search on non-radiographic growth predictors | Sex hormones in relation to pubertal growth velocity |
| 71. Shu et al., 2024 | Targeted supplementary search on sex hormones and skeletal maturity | Associations between sex hormones and bone age |
| 72. Kapoor et al., 2023 | Targeted supplementary search on biomarkers of skeletal maturity | Systematic review of body-fluid biomarkers |
| 73. Welborn et al., 2025 | Reviewer-identified source followed by bibliographic verification | Prospective multicenter evidence linking CXM to height velocity |

**Supplementary Figure S1. Flow of literature identification, manual screening, full-text assessment, and evidence selection for the narrative review**
